# New-onset restless leg syndrome in a COVID-19 patient: a case report with literature review

**DOI:** 10.11604/pamj.2021.38.318.28836

**Published:** 2021-03-30

**Authors:** Osama Mohiuddin, Anosh Aslam Khan, Syed Muhammad Ismail Shah, Mohammad Daniyal Zafar Malick, Shehzeen Fatima Memon, Sumeen Jalees, Farah Yasmin

**Affiliations:** 1 Department of Internal Medicine, Dow University of Health Sciences, Karachi, Pakistan,; 2 Department of Internal Medicine, Ziauddin Medical University, Karachi, Pakistan,; 3 Department of Neurology, Dow University of Health Sciences, Karachi, Pakistan

**Keywords:** Restless leg syndrome, COVID-19, sleep, case report

## Abstract

Restless leg syndrome (RLS) is a sleep disorder characterized by the sudden urge to move the lower limbs during periods of rest accompanied by an unpleasant sensation like tingling or burning in the legs. Often, this urge is partially relieved by the movement of legs. However, it causes disturbance of sleep leading to daytime fatigue. Herein, we present an unusual case of new-onset of restless leg syndrome in a COVID-19 infected patient who presented three weeks after an uncomplicated delivery via caesarean section. The patient was managed with sleep hygiene measures, oral iron and vitamin C tablets apart from general COVID-19 management medications, subsequently leading to significant improvements. Here we have discussed possible associated factors, pathophysiological mechanisms and management of RLS in the case of COVID infected individuals.

## Introduction

Since the global spread of COVID-19, millions of people have been infected. The spectrum of COVID-19 infection has been noted to range from asymptomatic to severe respiratory distress syndrome leading to potentially fatal pneumonia. Apart from pulmonary infection, a wide range of gastrointestinal and neurological manifestations have also been observed [1]. Moreover, the critical impact of COVID-19 on the psychological health of the people also gradually came to light after a series of global lockdown implementations began in the first quarter of 2020. This leads to an extended limitation on social interactions, physical activities and also created a profound sense of fear and anxiety among the general population [2]. Sleep is a fundamental physiological phenomenon that is also impacted by metabolic derangements, acute illnesses and mood changes. A study conducted by Karadas *et al*. showed that around 30% of the patients suffered from sleep disturbances during the active COVID-19 infectious period while 1.7% of the patients also had overlapping symptoms of restless leg syndrome (RLS) [3].

Restless Leg Syndrome (RLS) is a distressing sleep disorder with the prevalence ranging from 2.5% to 15% in the general population. It is characterized by the sudden urge to move the lower limbs during periods of rest accompanied by an unpleasant sensation like tingling or burning in the legs. These urge and sensation are often partially relieved by movements, however, they disturb the sleep quality leading to daytime fatigue and sleepiness. Furthermore, RLS can be classified as a primary disorder in cases of the idiopathic origin or it can occur secondary to diabetes mellitus, renal failure, iron deficiency, pregnancy and polyneuropathies [4]. Moreover, infections such as COVID-19 has also been noted to cause sleep disturbance and RLS by the complex interaction of immune response with the central nervous system [1]. Nevertheless, subjective factors can also play a role in the augmentation of symptoms of RLS. Herein, we describe a case of a 36-year-old, three weeks postpartum and COVID positive patient who developed new-onset restless leg syndrome during her hospitalization period. To the best of our knowledge, this is the first reported case of RLS in a COVID-19 patient from the developing country of Pakistan.

## Patient and observation

A 36-year-old female presented to the emergency department with a 4-days history of severe generalized myalgia, low-grade fever and cough. The cough was mild, dry and constant. The fever was constant which used to subside by taking antipyretics only. She was a non-smoker with no chronic history of co-morbidities except gestational diabetes which she developed during her latest pregnancy. Three weeks earlier, she had an uncomplicated delivery of her fourth child via Caesarean section. During her pregnancy, she developed gestational diabetes for the first time which was carefully managed through dietary control and insulin. Other than that, she had no history of chronic co-morbidities or smoking. There was no significant family history except for diabetes on both maternal and paternal side.

**Figure 1:**
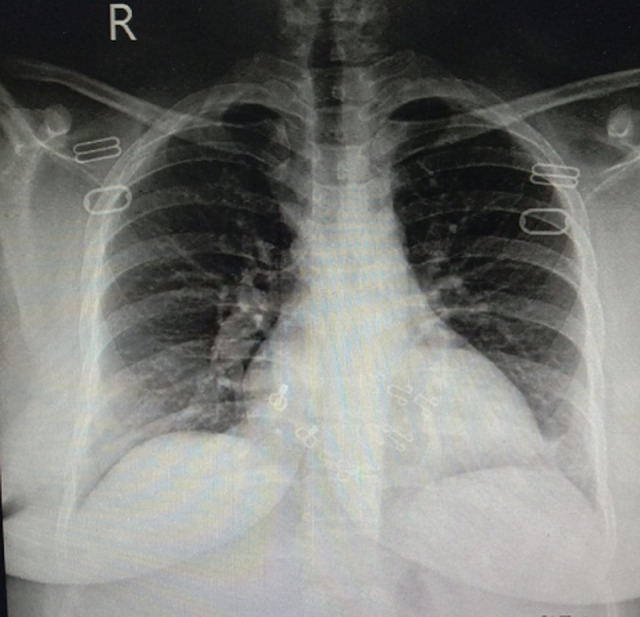
chest X-ray (AP view) showing perihilar prominence with bilateral interstitial infiltrate

Upon arrival, she was conscious and oriented with time, place and person with the respiratory rate of 26 breaths per minute, temperature 100°F, blood pressure 100/70 mmHg, pulse 96 beats per minute and arterial oxygen saturation (SpO2) was 92% at rest. On auscultation, bilateral coarse crepitation was audible throughout both the lung fields. Laboratory analysis revealed an elevated leukocyte count of 13 x 10^9^/L (normal 4-10 x 109/L) and a decreased hemoglobin level of 9.0 g/dl (normal 12.0 to 15.5 g/dl). The random blood glucose level was 225 mg/dL. Both of the erythrocyte sedimentation rate (ESR) and C-reactive protein (CRP) were elevated as follows: 42 mm/hr (normal: 0-20 mm/hr) and 123 mg/L (normal: < 5 mg/L), respectively. Lactate dehydrogenase (LDH) was also raised at 183 U/L. However, serum ferritin and d-dimers levels were within the reference range. To confirm the high suspicion of COVID-19, coronavirus nucleic acid test (NAT) via real-time fluorescence polymerase chain reaction (RT-PCR) was ordered, which came out positive. Henceforth, the chest X-ray was ordered (Figure 1) and the patient was admitted to the COVID-19 isolation ward on the day of the presentation.

A probable differential diagnosis of restless leg syndrome was made. A complete iron panel was ordered which showed normal ferritin level of 36 ng/mL, low serum iron level of 20 μg/dL (normal: 60-170 μg/dL), high total iron-binding capacity of 625 μg/dL (normal: 240-450 μg/dL) and low transferrin saturation of 5% (normal: 15%-50%). This confirmed the presence of iron deficiency anemia in the patient. Meanwhile, serum folate, vitamin B12, thyroid profile, serum electrolytes level were all within the reference range. It was decided to proceed with the polysomnography to study the sleep cycle, however, the patient was irritable and did not give her consent for the study despite multiple efforts. The patient was started with the additional medications of antiviral, antibacterial (lopinavir/ritonavir and piperacillin-sulbactam, respectively), 325 mg of oral ferrous sulphate and 500 mg vitamin C tablets. The patient was instructed to avoid any caffeinated drinks and do light limb massage during the night before sleep to improve sleep hygiene. This pharmacological and non-pharmacological routine was continued for the next five days which brought significant relief to the patient. After complete clinical improvement and two successive negative repeats of RT-PCR performed on the 10^th^ and 12^th^ hospital day, the patient was discharged with the prescription to continue vitamin C and iron supplements for 4-5 weeks.

## Discussion

### General characteristics of restless leg syndrome (RLS)

Restless leg syndrome (RLS) is a sensorimotor disorder characterized by uncomfortable sensations in the legs and the urge to move them. These symptoms are one of the four criteria for the diagnosis of RLS. The sensations could be described as itching bones, burning, throbbing, pulling, or crawling, etc, although they are difficult to describe and could be varied [5]. The other criteria include: 1) symptoms being worse in periods of rest and inactivity; 2) partially or completely relieved by movement, such as stretching or walking, at least till the activity is continued. 3) The symptoms also occur only during the night or worsen during the night or evening, if also present during the day.

Symptoms also tend to recur and can cause impaired sleep quality and daytime sleepiness. Point prevalence from Asian countries can be around 1.1 to 2.1%, although one study from Pakistan reported a point prevalence of 23.6% [4]. The prevalence is also higher in females. Higher incidence of iron deficiency anemia and low ferritin levels in females of the developing world could explain the female gender predisposition. Having a family history can cause early-onset disease. As such, RLS can occur at any age [4, 6].

### Possible genetic cause(s)

The risk of early onset of symptoms is usually before 45 years of age in patients with a first-degree relative. Certain phenotypes can be associated with the absence of family history in patients, such as the risk of more neuropathy, later age of onset, rapidly progressing symptoms, and having comorbid disorders [7]. The modes of inheritance described in the literature include both autosomal dominant and recessive, however many gene loci are involved in different populations. The high-activity allele for Monoamine oxidase, present only in females, can also increase the risk of RLS in women by lowering levels of synaptic dopamine [8].

### Exacerbating and associated factors of RLS

Secondary or exacerbating causes of RLS include iron deficiency anemia, diabetes, pregnancy, renal failure, or peripheral neuropathy [9]. Our patient had iron deficiency, was parous, and had high random blood sugar levels. Therefore, it was more likely a secondary case of resting leg syndrome. CSF ferritin levels are found to be lower in patients with RLS symptoms, however, in the serum, these proteins might not be good predictors of intracellular iron. In the brain, decreased ferritin and iron storage in neuromelanin cells be good predictors of the disease [7]. Ferritin is also an acute-phase reactant protein. According to a meta-analysis involving 10,614 COVID-19 confirmed patients, high levels of ferritin in COVID-19 could determine the severity of the disease [10]. Interestingly, it was not elevated in our patient.

Confinement due to COVID-19, and the inability to participate in pleasurable activities can lead to higher levels of stress, predisposing to more mood disorders such as anxiety and depression. Moderate and severe symptoms of RLS can result from having sympathetic hyperactivity, causing an increase in cortisol and subsequent awakening and excitation of movement. The resultant is a reduction and fragmentation of sleep, predisposing to more health risks [2]. Sleep disorders during the pandemic have also been found to be more common in females [11].

### Pathophysiology is multifactorial and interconnected

RLS dysfunction has been linked to subcortical dopaminergic systems, which are involved in the regulation of sleep [7]. The pathophysiology can be multifactorial; however, the involvement of the central nervous system is highly likely. This is because only the dopamine agonists or antagonists which can pass through the blood-brain barrier have been shown to affect the symptoms of RLS. In contrast, drugs like domperidone, which cannot pass through the blood-brain barrier have shown no effect on the symptoms. Therefore, mostly neuronal loops involving the cerebellum, thalamus, red nucleus, and inferior olive have been described to be involved. The nigrostriatal system (A9) is important due to its control of voluntary movement. The A11 system and its anatomical connection with the suprachiasmatic nucleus of the hypothalamus involved in the regulation of circadian rhythms is another postulation. The blood-brain barrier is also compromised in COVID-19 infection due to cytokines involved such as IL-1β, IL-6, TNFα, and IL-17. Cytokines in the CNS can cause microglial activation and proliferation (MAP), which have shown to disrupt endothelial tight junction [12]. Neurological dysfunction in COVID-19 can be seen as headaches, dizziness, myalgias, smell and gustation problems and cerebrovascular disorders. However, one study found 12.6% of their patients to have sleep disturbance, while a small number of them (1.7%) had RLS [3].

RLS symptoms in pregnancy usually appear in the third trimester, most likely attributed to the physiological increase in hormones such as estrogen, progesterone, and prolactin till the third trimester. Although our patients had delivered very recently, RLS symptoms usually disappear after delivery [13]. Uniquely in our case, they developed after her pregnancy. Still, her parity was seemingly a risk factor for the development of RLS three weeks later. Parity and RLS have certain common associated metabolic and autonomic dysfunctions [14]. Parity can predict early-onset hypertension, cardiovascular disease, and diabetes, etc, while RLS has been positively associated with these conditions. Importantly, physiological alterations in pregnancy can lead to insulin resistance, decreased glucose tolerance, fat accumulations, dyslipidemia, and weight gain, in addition to hemodynamic changes and vascular remodelling. These conditions can also persist in the post-pregnancy period [15]. There has also been no significant difference found between the prevalence of RLS in nulliparous women and men, which shows parity as a risk factor for RLS [7].

Similarly, thyroid-stimulating hormone (TSH) levels also increase during the evening, causing RLS symptoms to usually occur late in the day [6]. Dopamine can induce the cytochrome P450 (CYP450) enzymes for degradation of TSH. The CYP450 are heme enzymes, therefore, lower levels of iron can diminish their effect. Iron supplies are also low in end-stage renal disease and pregnancy, which is why they are important secondary causes of RLS. At the same time, iron deficiency can limit the rate-determining enzyme of dopamine synthesis, further confirming dopaminergic pathways to be involved [7]. Hepcidin production is also upregulated in the choroid plexus associated-ferroproteins due to the IL-6 mediated cytokine storm production in COVID-19. COVID-19 infection can further deplete iron stores, especially in the CNS [1]. Apart from the described endocrine and metabolic pathways above, immune-mediated disruption due to COVID infection is also possible. This is because an acute phase response is necessary for recovery. However, eventually, there is an inducement of the production of cortisol and epinephrine. According to our search, only one case report was found of COVID-19 infection in association with restless leg syndrome. This case was also of a woman with a previous history of cardiovascular disease and diabetes mellitus. Her transferrin levels were normal like in our patient. In contrast, inflammatory markers like ESR and C-reactive protein were elevated in our patient [1].

### Possible treatments and iron supplementation in case of COVID-19 inflammation

A connection between RLS and major depressive disorder can be established due to a dopaminergic pathology involved. Dopamine agonists are given in treatment-resistant depression or bipolar depression [16]. However, giving dopamine agonists (pramipexole and ropinirole) for RLS can be followed by worsening of the symptoms, which is not due to the natural progression of the disease. This is known as “Augmentation”. Patients receiving treatment for a year can start experiencing more intense symptoms earlier in the day, extending to more areas of the body [17]. Augmentation frequency was also found to be more with pramipexole treatment than with pregabalin. Pregabalin is an α2d ligand of the calcium channels. These ligands cause inhibition of the neurotransmitter glutamate in the CNS. Opioids such as oxycodone and methadone and benzodiazepines such as clonazepam are also used for RLS, however, more studies are needed to prove their efficacy [18].

Under chronic inflammation conditions like rheumatic heart disease, administration of iron has been argued to not be effective due to iron trapping within the macrophages and hepatocytes. This is manifested as higher levels of ferritin [19]. COVID-19 is also an inflammatory condition that can affect iron homeostasis [20]. However, iron supplementation has been seen to improve symptoms in patients with ferritin below 100 μg/L, which was true in our case. Vitamin C supplementation with oral forms of ferrous sulfate (325 mg, twice or thrice daily) can be administered [7]. Successful treatment can still cause patients to have sleep problems, probably due to classical conditioning [5].

### Limitations

One obvious limitation of this case study is the inability to determine the severity of the disease. One of the severity indexes is the use of the full polysomnogram, which can help provide a direct measure of the periodic limb movement in sleep (PLMS), seen in 90% of the patients of RLS [21]. Our patient however did not consent to the procedure. Repeated assessment over five nights can be provided with actigraphy, which can reduce concerns of variations in PLM rates every night. An RLS-sleep log reduces the likelihood of approximation of RLS events and establishes a detailed memory in the patient, however, compliance can be an issue. CSF analysis could also not be done, especially for ferritin levels. However, a probable diagnosis was made with the above investigations and her condition had improved with treatment.

## Conclusion

Although poor sleep quality is a common manifestation of COVID-19 infection, RLS is still an uncommon pathology. This mandates further research into the neurobiological aspect of COVID-19 on sleep disorders especially in terms of pathophysiology, preventive and management measures. It also essential for physicians and neurologists to keep COVID-19 as a potential trigger while managing RLS in a patient. Furthermore, other common exacerbating factors of RLS should also be observed and subsequently managed while treating with COVID-19 infection to improve their sleep and health quality.
